# Outcomes of an opioid reduction tool among patients with chronic noncancer pain in primary care

**DOI:** 10.1093/fampra/cmag049

**Published:** 2026-07-30

**Authors:** Loes de Kleijn, Elsemiek A W Jansen-Groot Koerkamp, Alessandro Chiarotto, Mette Heringa, Hanneke J B M Rijkels-Otters, Jeanet W Blom, Mattijs E Numans, Marcel L Bouvy, Bart W Koes

**Affiliations:** Department of General Practice, Erasmus MC, University Medical Center, Rotterdam 3015 GD, The Netherlands; SIR Institute for Pharmacy Practice and Policy, Leiden 2331 JE, The Netherlands; Division of Pharmacoepidemiology and Clinical Pharmacology, Utrecht Institute for Pharmaceutical Sciences, Utrecht University, Utrecht 3508 TB, The Netherlands; Department of General Practice, Erasmus MC, University Medical Center, Rotterdam 3015 GD, The Netherlands; SIR Institute for Pharmacy Practice and Policy, Leiden 2331 JE, The Netherlands; Department of General Practice, Erasmus MC, University Medical Center, Rotterdam 3015 GD, The Netherlands; Department Public Health and Primary Care, Leiden University Medical Center, Leiden 2300 RC, The Netherlands; Department Public Health and Primary Care, Leiden University Medical Center, Leiden 2300 RC, The Netherlands; Division of Pharmacoepidemiology and Clinical Pharmacology, Utrecht Institute for Pharmaceutical Sciences, Utrecht University, Utrecht 3508 TB, The Netherlands; Department of General Practice, Erasmus MC, University Medical Center, Rotterdam 3015 GD, The Netherlands; Department of Public Health & Center for Muscle and Joint Health, Research Unit of General Practice, University of Southern Denmark, Odense 5230, Denmark

**Keywords:** MESH terms: chronic pain, analgesics, opioids, general practice, harm reduction, guideline adherence

## Abstract

**Background:**

In a previous study, an opioid reduction tool was designed to reduce inappropriate opioid treatment in patients with chronic noncancer pain.

**Objectives:**

This study aimed to assess whether the tool facilitated opioid tapering in patients with chronic noncancer pain and long-term opioid treatment in primary care. In addition, it evaluated the impact on pain-related outcome measures.

**Methods:**

In a cohort study, 55 general practices and 30 collaborating pharmacies were recruited to implement the tool containing six reduction measures. Patients on long-term opioid treatment (>3 months) for chronic noncancer pain received a tailored taper programme from their healthcare provider and were monitored via questionnaires. The primary outcome was reduction in oral morphine equivalent daily dose over 3 months retrieved through pharmacy dispensing data. Secondary outcomes included trends in opioid use over 9 months, pain severity, pain interference, well-being, withdrawal symptoms, discontinuation rate, and nonopioid analgesic use.

**Results:**

Of the 366 patients approached, 116 agreed to taper, of whom 27 were included in our cohort. The median oral morphine equivalent daily dose decreased significantly from 40.0 mg (interquartile range (IQR), 16.5–120.0) at baseline to 15.2 mg (IQR, 0.0–60.0) at 3 months. Opioid dose continued to decrease over 9 months, with 12 participants discontinuing completely. Pain-related outcome measures remained stable throughout the study period.

**Conclusion:**

With a significant decrease in opioid dose without negative impact on pain, this study showed promising results for an opioid reduction tool tapering long-term opioid treatment in primary care. A larger controlled trial is needed to assess the effectiveness of the tool.

Key messages

1. Motivated patients on long-term opioid treatment can taper opioids in primary care.
2. Patient outcomes, such as pain and well-being, do not deteriorate during tapering.
3. Additional large, controlled implementation trial with improved tool needs to affirm results.

## Introduction

Chronic noncancer pain (CNCP) defined as pain not caused by cancer lasting for over 3 months, poses a significant burden on individuals, healthcare, and societies worldwide [1, 2]. CNCP not only affects physical functioning but also the overall quality of life [1]. In the management of CNCP, opioids have been widely prescribed, due to their perceived analgesic potency [3]. However, an increasing number of studies demonstrate limited benefits of long-term opioid treatment (LTOT) in CNCP [4, 5]. Additionally, studies reported side effects of opioids, including addiction, overdose, and opioid-induced hyperalgesia [4], as well as poor functional outcomes and increased healthcare utilization in patients on LTOT [6].

In recent years, a paradigm shift occurred in the management of CNCP, with most contemporary guidelines no longer recommending opioids as a first-line treatment [7, 8]. The UK's National Institute for Health and Care Excellence guideline [9] and the Dutch primary care guideline on pain [8] discourage the initiation of opioids in CNCP and recommend tapering for patients on LTOT. Implementing these recommendations in clinical practice, however, remains a challenging task [10, 11]. In fact, studies highlight that general practitioners (GPs) often find it difficult to initiate opioid taper conversations [11]. This is supported by qualitative studies among patients, describing absence of GP- or community pharmacist (CP)-led initiation of taper conversations [12]. In addition, proven opioid reduction strategies suitable for primary care are still lacking [13].

To address this research gap and assist primary care providers to implement pain guidelines, we previously developed an opioid reduction tool for primary care through a Delphi approach [14]. A 21-member multidisciplinary expert panel including two patients with lived experience decided on the tool's components by usefulness through voting and an in-depth discussion [14]. The developed tool comprises two parts. Part A included components that aimed to reduce opioid initiation and prevent long-term opioid use in noncancer pain management with results to be reported separately. This study aims to assess changes in opioid use after implementation of Part B of the tool, which aims to reduce long-term opioid use. It also evaluates the tool's impact on core outcome domains for CNCP to address concerns about increased pain, well-being, and withdrawal symptoms during tapering [15].

## Materials and methods

The Tool for reducing Inappropriate Opioid Use for Patients, Physicians and Pharmacists (TRIO-3P) cohort study with a pre- and posttest analysis conducted from September 2022 until June 2024 is reported following the STrenghtening the Reporting of OBservational studies in Epidemiology (STROBE) statement [16]. The protocol was prospectively registered in Open Science Framework (ID W3KS6; https://osf.io/w3ks6).

### Ethical approval

This study was exempt of formal ethical approval by the Medical Research Ethics Committee NedMEC based on the Dutch Medical Research Act (WMO) (reference number: 22/089). Nonetheless, the study protocol was reviewed and approved by the Institutional Review Board of the Division of Pharmacoepidemiology and Clinical Pharmacology, Utrecht University, with approval number UPF 2208.

### Participants

Fifty-five general practices and 30 affiliated community pharmacies were recruited for this study and conducted patient selection. GP practices were eligible only if their affiliated pharmacy also agreed to participate. Patients were eligible for inclusion if they met the following criteria: (i) diagnosed with CNCP for at least 6 months; (ii) receiving LTOT, defined as any daily opioid use for at least 3 months; (iii) aged 18 years or older; and (iv) sufficient Dutch proficiency to participate in the study. Patients receiving LTOT for cancer-related pain or methadone for treating illicit opioid dependence were excluded. Eligible patients received oral and written study information. Inclusion was confirmed after receiving a signed informed consent.

### Opioid reduction tool

The opioid reduction tool was designed as a minimal intervention strategy [17]. GPs and CPs received a 2-h academic detailing session on the opioid reduction tool provided by the main researchers (L.d.K. or E.A.W.J.-G.K.). The tool included the following: (i) five-step approach to identify, invite, motivate, and guide patients on LTOT, (ii) prewritten motivational invitation letter, (iii) motivational interview guide, (iv) tapering guide, (v) patient information folders on opioids and pain, and (vi) accredited e-learning on opioid reduction for all involved healthcare providers.

After formal inclusion, patients received a tailored opioid tapering schedule from their GP and/or CP. GPs and/or CPs were further instructed to discuss patients' needs, including involvement of a general practice mental health professionals and/or psycho-somatic physiotherapists during tapering. Throughout opioid tapering, GPs, CPs, and patients collaboratively decided on the pace of opioid tapering.

### Study procedures and data collection

At baseline, patients received a questionnaire with questions regarding socio-demographics, pain, opioid use, and personal (medical) history (Supplementary Appendix S1). Furthermore, patients were requested to complete surveys regarding their pain, pain interference, well-being, and withdrawal symptoms at baseline and three follow-up moments (1.5, 3, and 9 months). The surveys could be completed electronically via the software CASTOR EDC or sent by post. Medication dispensing data were obtained from participating pharmacies' records via the Dutch Foundation of Pharmaceutical Statistics (SFK) (Supplementary Appendix S1). In addition, participating GPs (one per practice) were requested to answer surveys regarding recruitment process and interdisciplinary collaboration at baseline and 6 months after the academic detailing session (Supplementary Appendix S1).

### Outcome measures

The primary outcome was the change in mean oral morphine equivalent (OME) daily dose dispensed from baseline to 3 months. Secondary outcomes included (i) OME trends from baseline to 9 months posttaper initiation; (ii) proportions of patients who successfully discontinued, reduced, or increased opioid treatment at 3, 6, and 9 months; (iii) proportions lost to follow-up at 1.5 and 3 months; (iv) changes in pain and pain interference [Brief Pain Inventory (BPI) scores]; (v) well-being [Beck Depression Inventory (BDI) scores]; (6) withdrawal symptoms [Subjective Opiate Withdrawal Scale (SOWS) scores] at baseline, 1.5, 3, and 9 months; and (7) additional use of nonopioid pain medication (Supplementary Appendix S1). The BPI is a validated self-report questionnaire that measures pain severity on a four-item pain intensity subscale (Likert scales 0–10) and pain interference on daily functioning on a seven-item pain interference subscale (Likert scales 0–10) [18]. The BDI is a validated self-report questionnaire that measures the level of depression (scores 1–63; 63 implying severe depression) [19]. Finally, the SOWS is a validated self-administered scale for grading 16 withdrawal symptoms on a 5-point Likert scale (scores 0–64; 64 implying severe withdrawal) [20].

### Data analysis

The primary outcome was calculated in a per-protocol analysis using a Wilcoxon signed-rank test due to non-normality. The OME daily dose in milligrams was calculated based on dispensing data, including opioids, starting with ATC code N02A using conversion factors as published by Nielsen *et al.* [21] (Supplementary Appendix S2). In addition, a sensitivity analysis, including a complete case analysis, excluding participants discontinuing the study preliminary was executed. Trend analysis of secondary outcomes was conducted using listwise deletion. Analysis of variance for repeated measures were applied to normally distributed BDI scores, and the nonparametric Friedman test was used for BDI, SOWS, and OME scores due to non-normality. Trends were presented descriptively using means with standard deviation (SD) or medians with interquartile ranges in case of non-normality. Other measures were presented descriptively using means, medians, and proportions as appropriate. To determine use of additional nonopioid pain relief drugs throughout opioid tapering, the proportion of patients with at least one nonopioid pain relief prescriptions in 30 days prior to all follow-up time points was determined. The following drugs (ATC code) were investigated using pharmacy dispense data: acetaminophen and combinations (N02BE), nonsteroidal anti-inflammatory drugs (NSAIDs) (M01), gabapentoids (N02BF), and antidepressants (N06A). Notably in the Netherlands, acetaminophen and low-dose NSAIDs are available over the counter, and consequently, the data may underestimate actual use. Normality of continuous data was tested using the Shapiro–Wilk test, and statistical significance was set at 5%.

## Results

### General practitioner participation and patient recruitment

**Figure 1:**
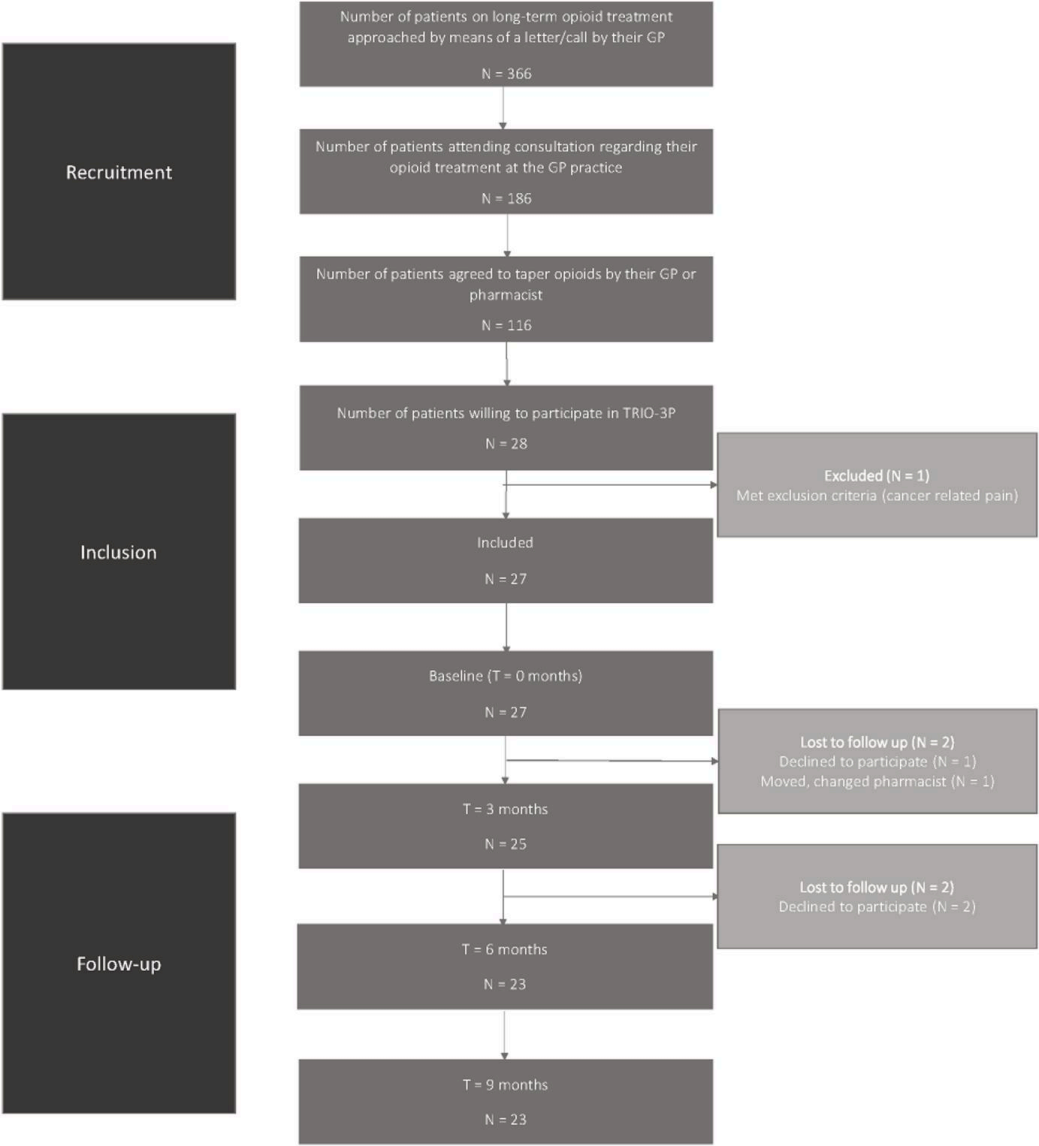
Flowchart TRIO-3P Part B patient recruitment, inclusion.

From September 2022 to February 2024, 46 participating general practices reported on recruitment through online surveys, indicating to have invited 366 patients for a taper consultation, with a median of five (range: 0–32) patients per general practice. A total of 186 patients (from 42 practices) agreed to discuss their LTOT during a consultation, with 116 patients (from 41 practices) ultimately agreeing to taper. According to GP surveys, pharmacies assisted with conceiving an opioid taper schedule in 16 (39.0%) general practices and 13 (31.7%) general practices reported to have referred patients for a taper consult at the pharmacy. Additionally, eight (19.5%) general practices referred patients for additional consultations to their nurse practitioner. The majority of the 116 patients did not agree to study participation, resulting in the inclusion of 27 patients of 15 participating general practices (range: 1–6 patient per practice) and 10 pharmacies (range: 1–9 patient per pharmacy) (Fig. 1). Participation refusal reasons were not documented.

### Patient demographics

Of 27 patients included in this study, 22 (82%) were female. The majority (N = 22) reported CNCP lasting for more than 5 years. The mean age was 60.7 years (SD ± 13.7), and they were prescribed a median OME daily dose of 40.0 mg (interquartile range (IQR), 16.5–120.0 mg) at baseline. Twenty patients reported having pain on more than one location. Most prevalent pain location was the back or neck (N = 20), followed by the hip or leg (N = 18). The mean BPI pain severity score at baseline was 6.2 (SD ± 1.9) on a numeric rating scale of 0–10. The majority of patients had tried to taper opioids at least once prior to this study (Table 1).

**Table 1:** Baseline characteristics of TRIO-3P Part B cohort.

Characteristic	Included participants
N	27
Female (*n*)	22
Mean age (years) (±SD)	60.7 (±13.7)
Median OME daily dose in mg (Q1–Q3)	40.0 mg (16.5–120.0)
Duration of pain (*n*)	
3 months–1 year	0
1–5 years	5
More than 5 years	22
Location of pain (*n*)	
Headache	2
Back or neck pain	20
Shoulder and arm pain	9
Hips and leg pain	18
Pain in hands	6
Pain in feet	9
Pain in multiple joints	9
Mean BPI pain severity (±SD)^a^	6.2 (±1.9)
Mean BPI interference(±SD)^b^	5.5 (±2.0)
Mean BDI score (±SD)^c^	12.3 (±7.8)
Median SOWS (Q1–Q3)^d^	11.0 (5.0–16.5)
Currently employed (*n*)	8
Net monthly income in Euro (*n*)	
<1500	10
1500–2500	4
>2500	5
Unknown	8
Currently receiving psychiatric treatment	4
History of prior opioid taper attempts (*n*)	15
Without guidance by healthcare provider	11
Guided by own GP	4
Smoking status (*n*)	
Current smoker	5
Former smoker	12
Never smoker	10
Current alcohol consumption (*n*)	
Yes	3
No	24
Current cannabis consumption (*n*)	
Yes	2
No	25

Q1 = 25th quartile, determined using Tukey's hinges, representing the data point that marks the 25th percentile, calculated as the median of the lower half of the dataset. Q3 = 75th quartile, determined using Tukey's hinges, representing the data point that marks the 75 percentiles, calculated as the median of the upper half of the dataset.

SD, standard deviation.

^a^BPI pain severity score based on four pain items of the BPI short form assessing pain at its ‘worst’, ‘least’, ‘average’, and ‘now’ on a scale of 0–10 from mild to severe pain.

^b^BPI interference score based on seven interference items of the BPI short form assessing how much pain has interfered with general daily activity, walking, work, mood, enjoyment of life, relations with others, and sleep on a scale of 0–10.

^c^BDI score based on the Dutch version, the BDI-II-NL, describing the severity of depressive complaints on a scale from 0 to 63 (normal to severe depressive symptoms).

^d^SOWS, based on the Dutch-validated version, with 16 items describing opiate withdrawal symptoms rated in intensity by patients on a 5-point scale of intensity (0 = not at all, 1 = a little, 2 = moderately, 3 = quite a bit, 4 = extremely) with the total score being the sum of all item ratings, ranging from 0 to 64 with higher scores for patients with more severe withdrawal symptoms.

### Patient participation

Of the 27 included patients, two patients dropped out at 2 months: one patient for undisclosed reasons and one patient moved to another nonparticipating pharmacy rendering opioid prescription data unavailable for analysis. Notably, the latter participant continued participation in online surveys. Two additional participants dropped out just after 3 months for unknown reasons. The patient survey response counts were 27 at baseline, 25 at 1.5 months, 21 at 3 months, and 19 at 9 months; 17 patients completed all surveys up to 9 months.

### Primary outcome

Comparing median OME daily dose at baseline [40.0 mg (IQR, 16.5–120.0)] with dose at 3 months [15.2 mg (IQR, 0.0–60.0)] of all included patients (N = 27) revealed a significant difference (Z, −3.773; *P* < .001). In a sensitivity analysis, excluding two participants who dropped out at 2 months, a smaller, but statistically significant, decrease in median OME daily dose (Z, −3.543; *P* < .001) was observed, with a baseline median (IQR) OME daily dose of 33.8 mg (15.9–117.5) and a median (IQR) OME daily dose of 16.5 mg (0.0–60.0) at 3 months.

### Secondary outcomes

#### Trend in oral morphine equivalent daily dose

**Figure 2:**
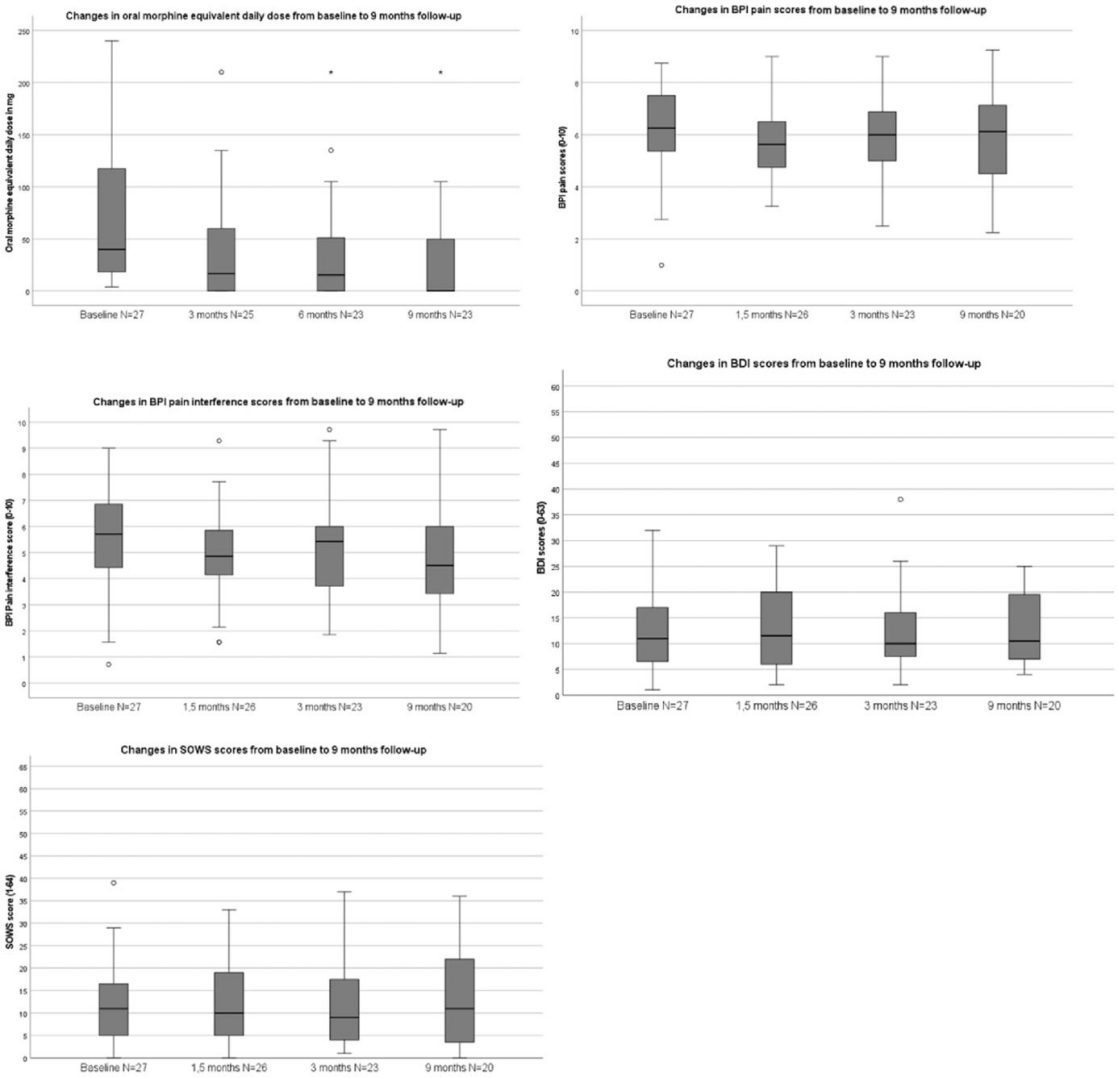
Changes in patient outcome measures across all follow-up time points.

The Friedman test, conducted on the 23 participants completing the study, demonstrated a significant decrease of OME daily dose across all follow-up time points (χ^2^, 38.682; *P* < .001) with a baseline median (Q1–Q3) OME daily dose of 40.0 (16.5–120) and a median (Q1–Q3) OME daily dose of 0.00 (0.0–60.0) at 9 months (Fig. 2; Supplementary Appendix S3).

#### Proportions of reduction and discontinuation

At 9 months of follow-up, 12 patients completely discontinued LTOT. With inclusion of these 12 patients, a total of 19 patients reduced their dose with at least 50% at 9 months (Table 2).

**Table 2:** Proportions of participants that reduced, discontinued, and increased LTOT^a^.

N = 23	T = 3 months	T = 6 months	T = 9 months
Reduction [*n* (%)]	16 (69.6)	17 (73.9)	20 (87.0)
50% reduction [*n* (%)]^b^	13 (56.5)	14 (60.9)	19 (82.6)
Discontinuation [*n* (%)]	8 (34.8)	9 (39.1)	12 (52.2)
Increase [*n* (%)]	1 (4.3)	0 (0)	0 (0)

^a^Based on a complete case analysis of 23 participants that completed the study.

^b^The reported proportions under ‘50% reduction’ include patients who discontinued opioid use, the aggregate of all four proportions does therefore not sum to 100%.

#### Trend in pain-related outcome measures

Analyses including 18 complete cases, determined that neither BPI severity [F (3, 51) = 0.541, *P* = .656] nor the BPI interference [F (3, 51) = 1.765, *P* = .166] differed significantly across time points. The BDI (χ^2^ = 0.476, *P* = .924) and SOWS (χ^2^  ^=^ 0.444, *P* = .931) scores also revealed no significant differences across time points (Fig. 2; Supplementary Appendix S3).

#### Use of nonopioid pain relief drugs

The dispense of at least one nonopioid pain relief drug among patients completing the study (N = 23) decreased over time from 15 (65.2%) at baseline to nine (39.1%) at 9 months of follow-up, including a large decrease in number of patients prescribed antidepressants between baseline and 9 months (Table 3).

**Table 3:** Proportions of participants who were dispensed concurrent nonopioid pain relief drug use.

(N = 23)	Baseline	T = 3 months	T = 6 months	T = 9 months
Acetaminophen [*n* (%)]	2 (8.7)	2 (8.7)	1 (4.3)	1 (4.3)
Nonsteroidal anti-inflammatory drug [*n* (%)]	4 (17.4)	5 (21.7)	5 (21.7)	4 (17.4)
Gabapentinoids [*n* (%)]	3 (13.0)	3 (13.0)	3 (13.0)	2 (8.7)
Benzodiazepines [*n* (%)]	3 (13.0)	0 (0)	5 (21.7)	3 (13.0)
Antidepressants [*n* (%)]	9 (39.1)	8 (34.8)	8 (34.8)	5 (21.7)
At least one of the above drugs [*n* (%)]	15 (65.2)	10 (43.5)	11 (47.8)	9 (39.1)

## Discussion

Among 27 participants, the OME daily dose significantly decreased at 3 months. At 9 months, 19 participants had reduced their opioid dose with at least 50%, of whom 12 participants fully discontinued LTOT. Secondary outcome measures demonstrated no changes in pain severity, pain interference, well-being, and withdrawal symptoms at 9 months.

As indicated by systematic reviews [13, 22], there is an urgent need for opioid reduction tools applicable in primary care to enhance guideline adherence, including tapering LTOT in patients with CNCP. Demonstrated by a substantial increase of opioid reduction studies performed in primary care [13, 23–25], it appears that this priority has been acknowledged in research. Most of these studies, however, propose resourceful reduction measures, including group therapies and multidisciplinary pain services [22]. Given the escalating healthcare costs, increasing workload, and growing shortage of healthcare personnel faced by GPs [26], there is an urgent need for minimal intervention strategies. To our knowledge, aside from one Australian study on additional patient support for opioid tapering using telephone text messaging [27] and one US study on the effect of GP-tailored opioid tapering [28], there are no other studies on less resource-intensive approaches to taper LTOT in patients with CNCP in primary care. Hence, this study being performed by GPs and CPs amidst their day-to-day practice contributes to an until now limited body of knowledge.

Although 12 of 23 patients discontinued LTOT, demonstrating that GPs, CPs, and patients can achieve opioid tapering using tool elements in routine primary care, the tool's implementability in everyday practice remains inconclusive due to the small sample size, largely attributable to the high proportion of patients who declined study participation. Across 55 general practices, 41 reported that 89 patients declined participation, but did, however, agree to taper their LTOT in primary care. Unfortunately, due to methodological constraints, we could not assess what proportion of these patients reached full discontinuation at the end of the study. A minimal intervention study tapering benzodiazepine performed in a similar setting in the Netherlands reached a 13% discontinuation rate after implementing a simple discontinuation letter to long-term users [17]. On the basis of these findings, the reported results may underestimate the tool's true impact on LTOT tapering. However, accurate measurement of this effect would require modifications to future study procedures. However, it is also plausible that the low inclusion rate reflects difficulty in adopting the tool within routine primary care and may therefore indicate limited implementability in real-world primary care settings. The planned mixed-methods evaluation is used to explore this possibility and, if appropriate, to reappraise and adapt tool components to better fit routine practice.

Various qualitative studies identified the anticipation of increased pain and decreased function as one important barrier to opioid tapering for both GPs and patients [11, 29]. By examining patient outcomes in the context of opioid tapering, this study addressed an important research priority by evaluating the legitimacy of this perceived barrier [30]. With no difference among participants in pain, pain interference, well-being, and withdrawal symptoms, this study adds to an increasing body of evidence suggesting that the anticipation of intensified pain and decreased well-being is not supported by empirical data [31, 32]. However, this and other studies lack statistical power due to low inclusion rates, making it imperative that larger controlled trials or systematic meta-analysis are conducted to confirm these findings. Ongoing controlled trials are expected to provide critical insights to address this research gap [33–35]. Additionally, studies should address whether patient's level of taper motivation affects measurements, such as pain. Nonetheless, our study results may already benefit GPs and CPs in taper conversations when addressing potential barriers, such as the anticipated increase in pain.

To further advance LTOT research, studies should also determine which patient subgroups benefit from minimal intervention strategies and which would need more resourceful opioid reduction methods. In addition, future research needs to explore which components of the opioid reduction tools explored in this study substantially impacted the LTOT taper success. A mixed-methods process evaluation of this study could inform necessary improvements and identify key reduction measures for GPs, CPs, and patients. Additionally, this evaluation should also examine whether the current division of responsibilities, where GPs have taken the lead in opioid tapering, is the most effective approach. For instance, a recent study (Perry, 2023) has shown that a stronger role for pharmacists can effectively contribute to opioid reduction in clinical practice [36]. This is not an isolated finding; multiple studies suggest that a more prominent proactive role by pharmacists could potentially strengthen opioid reduction programmes [37–39]. It is therefore worthwhile to further investigate whether shifting responsibilities in the implementation of this tool could further improve outcomes. Hence, it is by drawing on the forthcoming mixed-method evaluation, as well as on emerging evidence from controlled primary care opioid tapering studies, that the tool could be refined to further enhance its alignment with clinical practice, maximize its effectiveness, and subsequently be tested in its improved form. Beyond strengthening the opioid reduction tool, it is crucial to further explore whether sending a motivation letter alone can elicit outcomes comparable to those observed in studies on benzodiazepine tapering [40]. This is particularly relevant, considering the large number of patients who, after receiving the motivation letter attended a GP consultation and chose to taper LTOT.

### Strengths and limitations

This study is, to our knowledge, the first opioid taper study performed in Dutch primary care. One of the strengths of this study is its pragmatic and relatively simple design, demonstrating changes in outcomes in a manner that reflects its use in a typical, high-demand general practice and community pharmacy setting. The long follow-up of 9 months demonstrated the sustained changes in outcomes, extending well beyond the withdrawal phase.

With only 27 participants, statistical power is limited affecting the generalizability of the results. Moreover, due to low inclusion rate, we refrained from further sensitivity analysis on potential impact of additional pharmacist consultations or guidance by nurse practitioner and/or physiotherapist on the success rate of LTOT tapering. The high nonparticipation and low inclusion rates indicate potential barriers for both providers and patients. Unfortunately, reasons for nonparticipation were not systematically recorded. Potential barriers or explanations include substantial administrative burden for GPs associated with enrolment, participation burden for patients, exclusion of patients who had already self-tapered after receipt of the motivational letter, and withdrawal by patients who initially consented to tapering. Mitigation strategies for future controlled studies include enabling patient self-enrolment to reduce GP workload, limiting survey frequency, widening eligibility to include self-tapered patients, and extending follow-up to capture patients requiring more time to initiate tapering.

Additionally, the lack of a control group is an important limitation. The lack thereof resulted in the inability to correct for natural or spontaneous course of LTOT tapering as well as regression to the mean. Additionally, the process of patient selection and motivation to taper LTOT, carried out by CPs and GPs, may have introduced some degree of bias. However, the impact of this potential bias on the study's results is minimal, as this approach reflects routine clinical practice, ensuring the findings remain relevant and generalizable.

Finally, using dispense data to calculate OME daily dose involved additional limitations. Firstly, dispense data is potentially not equal to actual drug use. The OME daily dose presented could be underestimated when patients simultaneously used stockpiled opioids or overestimated if they used fewer opioids than were dispensed. Additionally, the calculation of the daily OME at 9 months can be overestimated. This is because the calculations at other time points incorporated future dispense data to distinguish between discontinuation and pseudo-drug holidays. However, due to the unavailability of dispense data after 9 months, this distinction could not be made for the calculation of the 9-month time point.

## Conclusion

This study contributes to a limited body of knowledge exploring minimal intervention strategies to taper LTOT in patients with CNCP in primary care. Although the study lacks statistical power due to the low participant inclusion, the reduction in OME daily dose at 3 months with no observed changes in patient outcomes, such as pain, is an encouraging result. A mixed-method process evaluation of this study and larger randomized controlled trials are necessary to substantiate our results.

## Supplementary Material

cmag049_Supplementary_Data

## Data Availability

The data underlying this article are available from the corresponding author upon reasonable request. Due to privacy and ethical considerations, only anonymized data can be shared.
