# Supplementary material for: Outcomes of an opioid reduction tool among patients with chronic noncancer pain in primary care
Source: Fam Pract. 2026 Jul 30;43(4):cmag049. doi: 10.1093/fampra/cmag049 (PMC13420516; doi:10.1093/fampra/cmag049)
Supplement: cmag049_Supplementary_Data [file cmag049_supplementary_data.pdf]

Appendix 1 Details on data collection

Drug dispensing data collection from participating patients:

Medication dispensing data for patients on LTOT for CNCP were retrieved from participating pharmacy records by the Dutch Foundation for Pharmaceutical Statistics (Stichting Farmaceutische Kerngetallen; SFK). Data covered all medications dispensed to each patient from the date of informed consent through 9 months thereafter and included: drug name, ATC code, dose, quantity dispensed, prescription start and end dates, prescribed daily amount, and cost information. From these dispensing records we extracted all opioid prescriptions (ATC code N02A) and non-opioid analgesics using ATC codes N02BE (paracetamol), M01 (anti-inflammatories and antirheumatic agents), N02BF (gabapentinoids) and N06A (antidepressants, including N06AX21 duloxetine).

Data collection through surveys

GP data collection

Each participating GP practice and pharmacy reported intervention use via electronic surveys administered in CASTOR EDC. One GP per practice and one pharmacist per pharmacy completed surveys about use and perceived usefulness of intervention components; those process (evaluations will be reported separately in a forthcoming evaluation paper). For the present manuscript we used data from the 6-month GP survey, including: number of LTOT patients invited for a tapering discussion, number of patients who had a tapering discussion, number of patients who agreed to taper LTOT, number of LTOT patients referred to secondary care for tapering, and number of tapering plans initiated with pharmacist support.

Patient data collection,

Participating patients who provided informed consent received surveys via CASTOR EDC or by post according to their preference. At baseline they completed a sociodemographic and brief medical-history survey that captured pain diagnosis, pain location(s), pain duration, medical history, and lifestyle factors. Specifically, respondents reported year of birth, gender, employment status and occupation, weekly work hours, monthly net income, education level, parents' country of birth, current and past psychiatric diagnoses and treatments, prior opioid tapering attempts and support, history of addiction/dependence (substances and treatments), and current and past smoking, alcohol, and illicit drug use (types and duration).

Patients were also asked to report analgesic medication use (opioid and non-opioid); however, patient feedback indicated the reporting table was too complex and medication-use data from this survey were therefore unreliable.

In addition to the baseline survey, patients completed the BPI, BDI, and SOWS at baseline and at 3 and 9 months.

The complete baseline survey (at baseline) and evaluation as sent to all participants is provided below:

Baseline Survey - Patients

Part 1: General questions

The first part consists of questions about:

- Your background
- Your pain complaints
- Your medical history
- Your lifestyle

Background

1. In which year were you born?
2. What is your gender? (male- female- I'd rather not say)
3. Are you currently working?
   - o Yes
   - o No, continue to question 6

4. What kind of work do you do?

1. How many hours do you work per week?

2. What is your estimated monthly net income*?

   ** Net income: the income you receive in your bank account each month*

   - o €0-1500,-
   - o €1500-2500,-
   - o €2500-3500,-
   - o More than €3500,-
   - o I'd rather not say

3. What is your highest degree degree?
   - o Primary school
   - o Secondary school (VMBO, HAVO, VWO)
   - o Vocational education (MBO)
   - o University of Applied Sciences (HBO)
   - o University
   - o Other, namely
4. What is your country of birth?
5. What is your father's country of birth?
6. What is your mother's country of birth?

Pain complaints

7. What was the indication for which you were prescribed opioids?*

**Opioids are strong painkillers such as tramadol, oxycodone (Oxycontin®/Oxynorm®), morphine, fentanyl (Instanyl®) and buprenorphine (BuTrans®).*

8. In which part(s) of your body do you have the pain for which you use opioids?

   Place an **X** on the area where you feel the most pain.

9. In which other place(s) do you have pain?
10. How long have you had this pain?

- o Less than 1 year
- o 1 to 5 years
- o More than 5 years

Medical history

11. Are you currently being treated by a psychiatrist, psychologist or an addiction specialist?
    - o Yes
    - o No, continue to question 17
12. If so, what is your diagnosis(s)? (tick the answer(s) that apply)
    1. Major depressive disorder
    2. Anxiety disorder
    3. Psychotic disorder (psychosis, schizophrenia)
    4. Personality disorder
    5. Substance abuse
    6. Other, namely
13. Have you been treated for a psychiatric illness in the past?
    - o Yes
    - o No, continue to question 19
14. If so, what was your diagnosis/were your diagnoses? (tick the answer(s) that apply)
    - o Major depressive disorder
    - o Anxiety disorder
    - o Psychotic disorder (psychosis, schizophrenia)
    - o Personality disorder
    - o Substance abuse
    - o Other, namely

15. Have you ever tried to taper off opioids before?

- o Yes
- o No, continue to question 22

16. If so, have you received guidance with tapering?

  - o Yes
  - o No

17. Who provided this guidance?

- o General practitioner
- o Pharmacist
- o Specialist
- o Other, namely

18. Have you ever been treated for an addiction to, or dependence on, any substance?

Think about alcohol, smoking and other medications.

- o Yes
- o No, continue to question 26

19. For which substance? (tick the answer(s) that apply)

    1. Tobacco (smoking)
    2. Alcohol
    3. Drugs
    4. Medicines
    5. Other, namely

20. If you have been treated for a medication addiction/dependence, which medication was it?

21. When were you treated for that?

Lifestyle

22. Do you smoke?

    1. Yes
    2. No, continue to question 28

23. If so, how many years have you been smoking currently?

24. Have you smoked in the past?

    - Yes
    - No, continue to question 30

25. If so, how many years have you smoked in total?

26. Do you drink alcohol?

    - Yes
    - No, continue to question 32

27. If so, how many glasses do you drink on average per week?

28. Do you use (illegal) drugs?

    - Yes
    - No, continue to question 35
    - I prefer not to answer this question, continue to question 35

29. If so, what drugs do you use? (tick the answer(s) that apply)

    - Cannabis
    - Ecstasy/MDMA
    - LSD
    - GHB
    - Cocaine
    - Heroin

- o Other, namely

30. How many years have you been using these drugs?

31. Have you used (illegal) drugs in the past?

    1. Yes
    2. No, continue to question 38
    3. I prefer not to answer this question, continue to question 38

32. If so, what drugs did you use? (tick the answer(s) that apply)

    1. Cannabis
    2. Ecstasy/MDMA
    3. LSD
    4. GHB
    5. Cocaine
    6. Heroin
    7. Other, namely

33. How many years have you used these drugs?

Part 2: Questions about your pain
The second part consists of questions about:

- Your pain
- Your pain management
- Hindrance due to pain

Pain

During our lives, most of us have experienced occasional pain (such as mild headaches, sprains, or toothaches).

34. Have you had any pain today that was different from these everyday types of pain?

- o Yes
- o No

Please rate your pain on a scale from 0 to 10. Here, 0 means no pain and 10 means the worst pain you can imagine. Circle the number that applies to you.

35. How much pain do you have when your pain is at its worst?

0 1 2 3 4 5 6 7 8 9 10

36. How much pain do you have when your pain is at its least?

0 1 2 3 4 5 6 7 8 9 10

37. How much pain have you had on average in the past 24 hours?

0 1 2 3 4 5 6 7 8 9 10

38. How much pain do you have right now?

0 1 2 3 4 5 6 7 8 9 10

Pain treatment

39. What opioids are you currently using? (tick which answer(s) apply)

1. Tramadol
2. Oxycodone
3. Fentanyl
4. Buprenorphine
5. Morphine
6. Methadone
7. Tapentadol
8. Other, namely

40. Are you using long-acting or short-acting opioids?

1. Long-acting
2. Short-acting
3. Both

41. Fill in the table below for the opioids you are using as in the example shown.

Example:

I use	*How many times?*	*Per*	*How much?*	*I use*	*How many milliliters?*	*I use this differently, namely:*
Fentanyl	*1*	*3 days*	*12 micrograms*	*Patches*	*n/a*	*n/a*

Which medicine do you use, how much, and in what form? For example: I use **oxycodone 3** times a **day 50 mg tablet**, or: I use **fentanyl 1** time every **3 days 12 microgram patches**.

Do you use the opioid differently? For example: As needed, 10 mg tablet 1 to 6 times a day, or: 1 tablet of 5 mg in the morning, 1 tablet of 5 mg in the afternoon, and 2 tablets of 5 mg in the evening.

Write that down in the last column of the table. **Now, fill in the table below.**

I use	*How many times?*	*Per*	*How much?*	*I use*	*How many milliliters?*	*I use this differently, namely:*

42. What other painkillers do you use? (tick the answer(s) that apply)

- o Paracetamol
- o NSAIDs*
- o Other, namely

** NSAIDs are drugs such as naproxen (Aleve®), diclofenac (Voltaren®) and ibuprofen (Brufen®).*

43. Fill in the table below for the other painkillers you use. Do this in the same way as the example in question 45.

I use	*How many times?*	*Per*	*How much?*	*I use*	*How many milliliters?*	*I use this differently, namely:*

44. Do you have any other treatments for your pain besides painkillers?

    (tick the answer(s) that apply)

    - Yes, I am being treated by a physiotherapist
    - Yes, I am being treated by a psychologist/psychiatrist
    - No
    - Other, namely

45. How much relief have medications and other pain treatments given you in the past 24 hours?

    Please choose a percentage between 0 and 100, where 0% means no relief and 100% means complete relief. (circle the percentage that applies)

    0% 10% 20% 30% 40% 50% 60% 70% 80% 90% 100%

Hindrance due to pain

For the following questions, choose the number that best describes how much your pain has limited your activities in the past 24 hours.

Here, 0 means no limitation and 10 means complete limitation. (circle the number that applies)

46. Your daily activities (think about showering, getting dressed, personal care, etc.)

    0 1 2 3 4 5 6 7 8 9 10

47. Your mood

    0 1 2 3 4 5 6 7 8 9 10

48. Your walking ability

    0 1 2 3 4 5 6 7 8 9 10

49. Your normal activities (this includes both work outside your home and household tasks)

0 1 2 3 4 5 6 7 8 9 10

50. Your relationships with other people

0 1 2 3 4 5 6 7 8 9 10

51. Your sleep

0 1 2 3 4 5 6 7 8 9 10

52. Your pleasure in life

0 1 2 3 4 5 6 7 8 9 10

Part 3: **Alternative pain treatment**

This section consists of questions about alternative pain treatments.

53. Are you currently using alternative or non-pharmacological

Pain treatments?

o Yes

o No

54. What alternative or non-pharmacological pain treatments do you use?

(tick the answer(s) that apply)

1. Acupuncture/massages
2. Meditation
3. Mindfulness/ACT*
4. Other, namely

** ACT: Acceptance and commitment therapy is a form of psychotherapy.*

Part 4: Questions about your mood

The next section consists of 22 questions about your mode.

For each item, tick the statement that best describes how you have felt in the **past two weeks**.

59.

- I don't feel sad
- I feel sad
- I'm constantly sad and I can't get it off my mind
- I'm so sad or unhappy that I can't bear it anymore

60.

- I'm not particularly discouraged about the future
- I am discouraged about the future
- I feel like I have nothing to look forward to
  - I feel like the future is hopeless and there is no chance of improvement

61.

- I don't feel like a failure
  - I feel like I've done something wrong more often than an average person
- When I look back over my life, all I see is lots of failures
- I feel like I'm a complete failure as a person

62.

- I enjoy everything just as much as I used to
- I don’t enjoy things as much as I used to
- I don't find real satisfaction in anything anymore
- I no longer have satisfaction from anything; I find everything anoying

63.

- I don't feel particularly guilty

- o I often feel guilty
- o I feel guilty most of the time
- o I feel guilty all the time

64.

- o I don't feel like I'm being punished for anything
- o I have the feeling that I will be punished again
- o I expect to be punished
- o I feel like I'm being punished now

65.

- o I don't feel disappointed in myself
- o I'm disappointed in myself
- o I'm disgust myself
- o I hate myself

66.

- o I don't feel like I'm worse than anyone else
- o I criticize myself for my weaknesses or mistakes
- o I keep blaming myself for my faults
- o I blame myself for everything bad that happens

67.

- o I am absolutely not considering ending my life
    - o I sometimes consider ending my life, but I would never do that
- o I would like to end my life
- o I would end my life if I had the chance

68.

- I don't cry more than usual
- I cry more now than I used to
- I cry constantly now
- I used to be able to cry, but now I can't anymore, even if I want to

69.

- I don't get annoyed more than usual
- I get annoyed or irritated more quickly than I used to
- I'm constantly annoyed these days
- I am no longer annoyed at all by things that used to annoy me

70.

- I have not lost interest in other people
- I am less interested in other people now than I used to be
- I have mostly lost interest in other people
- I have completely lost interest in other people

71.

- I make decisions just as easily now as I used to
- I procrastinate on making decisions more than I used to
- I have more difficulty making decisions
- I can't make decisions at all anymore

72.

- I don't feel like I look less good than I used to
- I worry about looking old and unattractive

  - I feel like my appearance has changed permanently, making me look unattractive
- I believe I look ugly

73.

- I can do my work about as well as I used to
- It takes extra effort get started on anything
- I really have to force myself to do anything
- I am no longer capable of doing anything at all

74.

- I sleep as well as usual
- I don't sleep as well as I used to
  - I wake up one to two hours earlier than usual in the morning and have difficulty getting back to sleep
- I wake up hours earlier than I used to and can't get back to sleep

75.

- I don't get tired faster than usual
- I get tired quicker than I used to
- I get tired of almost everything I do
- I'm too tired to do anything

76.

- I don't have less appetite than usual
- I have less appetite than I used to
- I have much less appetite than I used to
- I have no appetite at all anymore

77.

- o I have hardly lost any weight recently
- o I have lost more than 2 kilos, continue to question 78
- o I have lost more than 4 kilos, continue to question 78
- o I have lost more than 6 kilos, continue to question 78

78.

I try to lose weight by eating less

- o Yes
- o No

79.

- o I don't worry about my health any more than usual
  - o I worry about physical problems, e.g. if I feel pain somewhere, if my stomach is upset, if have constipation etc.
  - o I worry a lot about my physical problems and find it hard to think about anything else
  - o I worry so much about my physical problems that I can't think of anything else

80.

- o I have not noticed any recent change in my interest in sex
- o I am less interested in sex than I used to be
- o I am much less interested in sex now
- o I have lost interest in sex completely

Part 5: Questions about other (physical) complaints

In the next section, you will be asked to indicate the extent to which you suffer from other (physical) complaints. Note: When filling in the form, it is about how you have felt in the **last ten minutes.**

Tick the correct answer. Don't think too long, there are no wrong answers.

81. I feel anxious
    - o Not at all o A little bit o Quite o Quite a lot o Very
82. I feel like I have to yawn
    - o Not at all o A little bit o Quite o Quite a lot o Very
83. I perspire (sweat)
    - o Not at all o A little bit o Quite o Quite a lot o Very
84. My eyes are watering
    - o Not at all o A little bit o Quite o Quite a lot o Very
85. I have a runny nose
    - o Not at all o A little bit o Quite o Quite a lot o Very
86. I Have goosebumps
    - o Not at all o A little bit o Quite o Quite a lot o Very
87. I'm shaking
    - o Not at all o A little bit o Quite o Quite a lot o Very
88. I feel suddenly hot
    - o Not at all o A little bit o Quite o Quite a lot o Very
89. I feel suddenly cold
    - o Not at all o A little bit o Quite o Quite a lot o Very

90. My bones and muscles hurt

o Not at all o A little bit o Quite o Quite a lot o Very

91. I feel restless

o Not at all o A little bit o Quite o Quite a lot o Very

92. I feel sick

o Not at all o A little bit o Quite o Quite a lot o Very

93. I feel like I want to vomit

o Not at all o A little bit o Quite o Quite a lot o Very

94. My muscles are twitching

o Not at all o A little bit o Quite o Quite a lot o Very

95. I have a stomach cramp

o Not at all o A little bit o Quite o Quite a lot o Very

96. I feel like I need to use (opioids)

o Not at all o A little bit o Quite o Quite a lot o Very

Thank you very much for completing the survey!

Evaluation Survey - Patients

Part 1: Questions about your pain

The second part consists of questions about:

- Your pain
- Your pain management
- Hindrance due to pain

Pain

During our lives, most of us have experienced occasional pain (such as mild headaches, sprains, or toothaches).

1. Have you had any pain today that was different from these everyday types of pain?
   - o Yes
   - o No

Please rate your pain on a scale from 0 to 10. Here, 0 means no pain and 10 means the worst pain you can imagine. Circle the number that applies to you.

55. How much pain do you have when your pain is at its worst?

0 1 2 3 4 5 6 7 8 9 10

56. How much pain do you have when your pain is at its least?

0 1 2 3 4 5 6 7 8 9 10

57. How much pain have you had on average in the past 24 hours?

0 1 2 3 4 5 6 7 8 9 10

58. How much pain do you have right now?

0 1 2 3 4 5 6 7 8 9 10

Pain treatment

59. What opioids are you currently using? (tick which answer(s) apply)

    1. Tramadol
    2. Oxycodone
    3. Fentanyl
    4. Buprenorphine
    5. Morphine
    6. Methadone
    7. Tapentadol

8. Other, namely

60. Are you using long-acting or short-acting opioids?

1. Long-acting
2. Short-acting
3. Both

61. Fill in the table below for the opioids you are using as in the example shown.

Example:

I use	*How many times?*	*Per*	*How much?*	*I use*	*How many milliliters?*	*I use this differently, namely:*
Fentanyl	*1*	*3 days*	*12 micrograms*	*Patches*	*n/a*	*n/a*

Which medicine do you use, how much, and in what form? For example: I use **oxycodone 3** times a **day 50 mg tablet**, or: I use **fentanyl 1** time every **3 days 12 microgram patches**.

Do you use the opioid differently? For example: As needed, 10 mg tablet 1 to 6 times a day, or: 1 tablet of 5 mg in the morning, 1 tablet of 5 mg in the afternoon, and 2 tablets of 5 mg in the evening.

Write that down in the last column of the table. **Now, fill in the table below.**

I use	*How many times?*	*Per*	*How much?*	*I use*	*How many milliliters?*	*I use this differently, namely:*

62. What other painkillers do you use? (tick the answer(s) that apply)

- o Paracetamol
- o NSAIDs*
- o Other, namely

** NSAIDs are drugs such as naproxen (Aleve®), diclofenac (Voltaren®) and ibuprofen (Brufen®).*

63. Fill in the table below for the other painkillers you use. Do this in the same way as the example in question 45.

I use	*How many times?*	*Per*	*How much?*	*I use*	*How many milliliters?*	*I use this differently, namely:*

64. Do you have any other treatments for your pain besides painkillers?

(tick the answer(s) that apply)

- o Yes, I am being treated by a physiotherapist
- o Yes, I am being treated by a psychologist/psychiatrist
- o No
- o Other, namely

65. How much relief have medications and other pain treatments given you in the past 24 hours?

Please choose a percentage between 0 and 100, where 0% means no relief and 100% means complete relief. (circle the percentage that applies)

0% 10% 20% 30% 40% 50% 60% 70% 80% 90% 100%

Hindrance due to pain

For the following questions, choose the number that best describes how much your pain has limited your activities in the past 24 hours.

Here, 0 means no limitation and 10 means complete limitation. (circle the number that applies)

66. Your daily activities (think about showering, getting dressed, personal care, etc.)

0 1 2 3 4 5 6 7 8 9 10

67. Your mood

0 1 2 3 4 5 6 7 8 9 10

68. Your walking ability

0 1 2 3 4 5 6 7 8 9 10

69. Your normal activities (this includes both work outside your home and household tasks)

0 1 2 3 4 5 6 7 8 9 10

70. Your relationships with other people

0 1 2 3 4 5 6 7 8 9 10

71. Your sleep

0 1 2 3 4 5 6 7 8 9 10

72. Your pleasure in life

0 1 2 3 4 5 6 7 8 9 10

Part 2: **Alternative pain treatment**

This section consists of questions about alternative pain treatments.

73. Are you currently using alternative or non-pharmacological

Pain treatments?

- o Yes
- o No

74. What alternative or non-pharmacological pain treatments do you use?

(tick the answer(s) that apply)

1. Acupuncture/massages
2. Meditation
3. Mindfulness/ACT*
4. Other, namely

** ACT: Acceptance and commitment therapy is a form of psychotherapy.*

Part 3: Questions about your mood

The next section consists of 22 questions about your mode.

For each item, tick the statement that best describes how you have felt in the **past two weeks**.

22.

- o I don't feel sad
- o I feel sad
- o I'm constantly sad and I can't get it off my mind
- o I'm so sad or unhappy that I can't bear it anymore

23.

- o I'm not particularly discouraged about the future
- o I am discouraged about the future
- o I feel like I have nothing to look forward to
- o I feel like the future is hopeless and there is no chance of improvement

24.

- o I don't feel like a failure
- o I feel like I've done something wrong more often than an average person
- o When I look back over my life, all I see is lots of failures
- o I feel like I'm a complete failure as a person

25.

- o I enjoy everything just as much as I used to
- o I don’t enjoy things as much as I used to
- o I don't find real satisfaction in anything anymore

- I no longer have satisfaction from anything; I find everything anoying

26.

- I don't feel particularly guilty
- I often feel guilty
- I feel guilty most of the time
- I feel guilty all the time

27.

- I don't feel like I'm being punished for anything
- I have the feeling that I will be punished again
- I expect to be punished
- I feel like I'm being punished now

28.

- I don't feel disappointed in myself
- I'm disappointed in myself
- I'm disgust myself
- I hate myself

29.

- I don't feel like I'm worse than anyone else
- I criticize myself for my weaknesses or mistakes
- I keep blaming myself for my faults
- I blame myself for everything bad that happens

30.

- I am absolutely not considering ending my life

- I sometimes consider ending my life, but I would never do that
- I would like to end my life
- I would end my life if I had the chance

31.

- I don't cry more than usual
- I cry more now than I used to
- I cry constantly now
- I used to be able to cry, but now I can't anymore, even if I want to

32.

- I don't get annoyed more than usual
- I get annoyed or irritated more quickly than I used to
- I'm constantly annoyed these days
- I am no longer annoyed at all by things that used to annoy me

33.

- I have not lost interest in other people
- I am less interested in other people now than I used to be
- I have mostly lost interest in other people
- I have completely lost interest in other people

34.

- I make decisions just as easily now as I used to
- I procrastinate on making decisions more than I used to
- I have more difficulty making decisions
- I can't make decisions at all anymore

35.

- I don't feel like I look less good than I used to
- I worry about looking old and unattractive
- I feel like my appearance has changed permanently, making me look unattractive
- I believe I look ugly

36.

- I can do my work about as well as I used to
- It takes extra effort get started on anything
- I really have to force myself to do anything
- I am no longer capable of doing anything at all

37.

- I sleep as well as usual
- I don't sleep as well as I used to
- I wake up one to two hours earlier than usual in the morning and have difficulty getting back to sleep
- I wake up hours earlier than I used to and can't get back to sleep

38.

- I don't get tired faster than usual
- I get tired quicker than I used to
- I get tired of almost everything I do
- I'm too tired to do anything

39.

- I don't have less appetite than usual
- I have less appetite than I used to
- I have much less appetite than I used to
- I have no appetite at all anymore

40.

- I have hardly lost any weight recently
- I have lost more than 2 kilos, continue to question 41
- I have lost more than 4 kilos, continue to question 41
- I have lost more than 6 kilos, continue to question 41

41. I try to lose weight by eating less

- Yes
- No

42.

- I don't worry about my health any more than usual
- I worry about physical problems, e.g. if I feel pain somewhere, if my stomach is upset, if have constipation etc.
- I worry a lot about my physical problems and find it hard to think about anything else
- I worry so much about my physical problems that I can't think of anything else

43.

- I have not noticed any recent change in my interest in sex
- I am less interested in sex than I used to be
- I am much less interested in sex now

- o I have lost interest in sex completely

Part 5: Questions about other (physical) complaints
In the next section, you will be asked to indicate the extent to which you suffer from other (physical) complaints. Note: When filling in the form, it is about how you have felt in the **last ten minutes.**

Tick the correct answer. Don't think too long, there are no wrong answers.

44. I feel anxious
    - o Not at all o A little bit o Quite o Quite a lot o Very
45. I feel like I have to yawn
    - o Not at all o A little bit o Quite o Quite a lot o Very
46. I perspire (sweat)
    - o Not at all o A little bit o Quite o Quite a lot o Very
47. My eyes are watering
    - o Not at all o A little bit o Quite o Quite a lot o Very
48. I have a runny nose
    - o Not at all o A little bit o Quite o Quite a lot o Very
49. I Have goosebumps
    - o Not at all o A little bit o Quite o Quite a lot o Very
50. I'm shaking
    - o Not at all o A little bit o Quite o Quite a lot o Very
51. I feel suddenly hot
    - o Not at all o A little bit o Quite o Quite a lot o Very
52. I feel suddenly cold
    - o Not at all o A little bit o Quite o Quite a lot o Very
53. My bones and muscles hurt
    - o Not at all o A little bit o Quite o Quite a lot o Very
54. I feel restless
    - o Not at all o A little bit o Quite o Quite a lot o Very
55. I feel sick
    - o Not at all o A little bit o Quite o Quite a lot o Very
56. I feel like I want to vomit
    - o Not at all o A little bit o Quite o Quite a lot o Very
57. My muscles are twitching
    - o Not at all o A little bit o Quite o Quite a lot o Very
58. I have a stomach cramp

- o Not at all o A little bit o Quite o Quite a lot o Very

59. I feel like I need to use (opioids)
    - o Not at all o A little bit o Quite o Quite a lot o Very

Thank you very much for completing the survey!

Appendix 2 Calculation of Daily dose OME in mg using Pharmacists' dispensing data

The Oral Morphine Equivalent (OME) daily dose, measured in milligrams, of all opioid prescriptions of the participating patients was determined using the pharmacy dispense data disclosed by the Dutch Foundation for Pharmaceutical Statistics (SFK). The opioid prescriptions were identified using the Anatomical Therapeutic Chemical (ATC) code "N02A" which includes all opioid types. The OME daily dose was calculated by multiplying units per day according to the prescribed dosing regimen with the prescribed strength and the corresponding conversion factor as published by Nielsen et al[27]. In case of missing dosing regimens, the regimen was assumed from previous prescriptions. In the absence of available dosing regimen data, a hypothetical dosing regimen was calculated, assuming a prescription duration of 30 days. For prescriptions with a "as needed" regimen the average dosing unit per day determined on the full length of the prescription was used in the calculations of the OME daily dose.

The OME at baseline and at successive follow-up time points (i.e. T=3 months, T=6 months and T= 9 months) was calculated by identifying the OME daily dose of the most recent prescription dispensed within the 30 days preceding the relevant time point. A 30-day retrospective period was employed to address pseudo drug holidays resulting from stockpiled drugs. In case of multiple prescriptions dispensed in these 30 days, we assumed concurrent use if the additional prescription would have > 75% overlap with the last dispensed prescription. The OME daily dose of concurrent prescriptions were summed. In case, the overlap with the last dispensed prescription was less than 75%, it was inferred that the last dispensed opioid prescription represented an early refill and OME daily dose of the previous prescriptions were not summed. To distinguish between complete discontinuation and pseudo drug holidays, we examined the subsequent 90-day period, in case the last prescription identified within the 30 days prior to the relevant time point had officially concluded before that time point. If no opioid prescriptions were dispensed during these 90 subsequent days, full discontinuation was presumed. And the end date of the last prescription found in the 30 days prior to the time point was determined as the discontinuation date. If no opioid prescriptions were identified in the 30 days prior to the designated time point, full discontinuation of opioid use was assumed and the OME of the relevant time point was set at zero milligrams. For calculation of the OME daily dose at 9 months, prescription data of the subsequent 90-days period was unavailable, therefore, we assumed continued use in all patients that had a valid prescription up to 30 days prior to the 9-month time point and assumed continuation of MEDD of the last valid prescription. See attached figure for calculation examples.

Figure with calculation examples

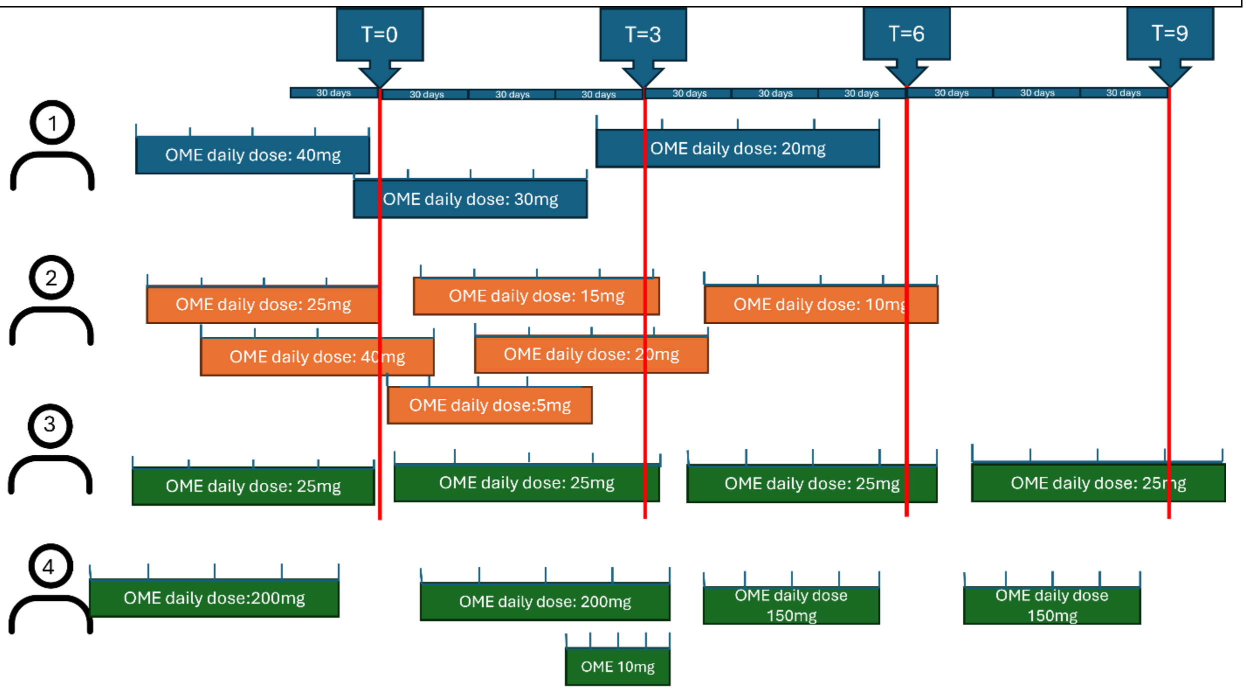

Calculation examples:

Participant 1:

- T=0 in 30 days prior to T=0 2 prescriptions. Overlap < 75% -> **OME daily dose =30mg**
- T=3 in 30 days prior to T=3 2 prescriptions. Overlap < 75% -> **OME daily dose =20mg**
- T=6 in 30 days prior to T=6 1 prescription. In subsequent 90 days no prescriptions, full discontinuation. Last prescription end date at 170 days -> **OME daily dose = 0 mg**
- T=9 no prescription in 30 days prior -> full discontinuation -> **OME daily dose = 0 mg**

Participant 2

- T=0 in 30 days prior to T=0 2 prescriptions. Overlap 75% -> **OME daily dose = 65 mg (40 +25)**
- T=3 in 30 days prior to T=3 3 prescriptions. Overlap 75% -> **OME daily dose = 40mg (15 +20 + 5)**
- T=6 in 30 days prior to T=6 2 prescriptions. Overlap < 75% -> **OME daily dose** and Subsequent 90 days no other prescription, i.e. full continuation after last prescription, end date at 195 days-> **OME daily dose = 10mg**
- T=9 no prescription in 30 days prior -> full discontinuation -> **OME daily dose = 0 mg**

Participant 3

- T=0 in 30 days prior to T=0 1 prescription. At T=0 no prescription “active”, looking forward 90 days, >1 prescription. T=0 based on last prescription in 30 days prior; time point T=0 falls within presumed pseudo drug holiday. -> **OME daily dose = 25 mg**
- T=3 in 30 days prior to T=3 1 prescription. -> **OME daily dose = 25 mg**
- T=6 in 30 days prior to T=6 1 prescription. -> **OME daily dose = 25 mg**
- T=6 in 30 days prior to T=6 1 prescription. -> **OME daily dose = 25 mg**

Participant 4

- T=0 in 30 days prior to T=0 1 prescription. At T=0 no prescription “active”, looking forward 90 days, >1 prescription. T=0 based on last prescription in 30 days prior; time point T=0 falls within presumed pseudo drug holiday. → **OME daily dose = 200mg**
- T= 3 in 30 days prior to T=3 2 prescriptions. Overlap 75% -> **OME daily dose = 210mg (10 +200)**
- T= 6 in 30 days prior to T=6 1 prescription. At T=6 no prescription “active”, looking forward 90 days, >1 prescription. T=6 based on last prescription in 30 days prior; time point T=6 falls within presumed pseudo drug holiday. → **OME daily dose = 150mg**
- T=9 in 30 days prior to T=9 1 prescription. At T=9 no prescription “active”, no dispense data in 90 days after T=9. Assuming continuation of last dispensed prescription and prescriptions with 75% overlap with this last prescription. → **OME daily dose = 150mg**

Appendix 3: Detailed summary of primary and secondary outcome measures

Table 4: Detailed summary of primary and secondary outcome measures				
**Secondary outcome measure**	**Time points**	**Mean score (± SD) or Median (Q1, Q3)**	**Participants(N)**	**Missing (N)**
**OME daily dose in mg**	Baseline	40.0 (16.5, 120.0)	27	0
	T = 3 months	20.0 (0.0, 60.0)	25	2
	T = 6 months	15.2 (0.0, 52.5)	23	4
	T = 9 months	0.0 (0.0, 60.0)	23	4
**BPI severity****	Baseline	6.2 (± 1.9)	27	0
	T = 1,5 months	5.8 (± 1.6)	26	1
	T = 3 months	5.8 (± 1.6)	23	4
	T = 9 months	5.9 (± 1.8)	20	7
**BPI interference score*****	Baseline	5.5 (± 2.0)	27	0
	T = 1,5 months	4.8 (± 1.8)	26	1
	T = 3 months	5.1 (± 2.0)	23	4
	T = 9 months	4.8 (± 2.1)	20	7
**BDI score******	Baseline	12.3 (±7.8)	27	0
	T = 1,5 months	13.0 (±7.4)	26	1
	T = 3 months	11.5 (±6.0)	22	5*
	T = 9 months	12.7 (±7.4)	20	7
**SOWS score*******	Baseline	11.0 (5.0, 17.0)	27	0
	T = 1,5 months	12.1 (± 9.2)<br>10.0 (4.8, 19.0)	26	1
	T = 3 months	9.0 (3.0, 18.0)	23	4
	T = 9 months	13.6 (± 11)<br>11.0 (3.3, 23.0)	20	7

SD = standard deviation. Q1 = 25th quartile, determined using Tukey's hinges, representing the data point that marks the 25th percentile, calculated as the median of the lower half of the dataset. Q3 = 75th quartile, determined using Tukey's hinges, representing the data point that marks the 75 percentile, calculated as the median of the upper half of the dataset.*Due to the non-normality of the data, an outlier was excluded from this descriptive analysis of de BDI at T=3 months to ensure the robustness and validity of the results presented in this table.** Brief Pain Inventory pain severity score based on four pain items of the BPI short form assessing pain at its “worst”, “least”, “average” and “now” on a scale 0-10 from mild to severe pain.*** Brief Pain Inventory interference score based on seven interference items of the BPI short form assessing how much pain has interfered with general daily activity, walking, work, mood, enjoyment of life, relations with others and sleep on a scale 0-10.**** Becks depression Inventory (BDI) score based on the Dutch version, the BDI-II-NL, describing the severity of depressive complaints on a scale from 0-63 (normal to severe depressive symptoms).***** Self-administered Opiate Withdrawal Scale (SOWS), based on the Dutch validated version, with 16 items describing opiate withdrawal symptoms rated in intensity by patients on a 5 point scale of intensity (0= not at all, 1 = a little, 2 = moderately, 3 = quite a bit, 4 = extremely) with the total score being the sum of all item ratings, ranging 0-64 with higher scores for patients with more severe withdrawal symptoms.
